# Supplementary material for: Genetic Landscape of Congenital Cataracts in a Swiss Cohort: Addressing Diagnostic Oversights in Nance–Horan Syndrome
Source: Biomedicines. 2025 Aug 2;13(8):1883. doi: 10.3390/biomedicines13081883 (PMC12383364; doi:10.3390/biomedicines13081883)
Supplement: Supplementary file 1 [file biomedicines-13-01883-s001.zip › biomedicines-3705163-supplementary.pdf]

Pedigrees Family 1 to 10:

Family 1:

ID	Gene	Cataract surgery timing	Cataract phenotype	Ocular and/or systemic abnormalities	2° glaucoma	VA (Snellen)
1-2	CRYBA1	6y	Nuclear	N/A	no	BE: 0.8
2-2	CRYBA1	1y9m	Nuclear	N/A	no	RE: 0.63<br>LE: 0.4
2-3	CRYBA1	10m	Nuclear	N/A	yes (LE)	RE: 1.0<br>LE: 0.5

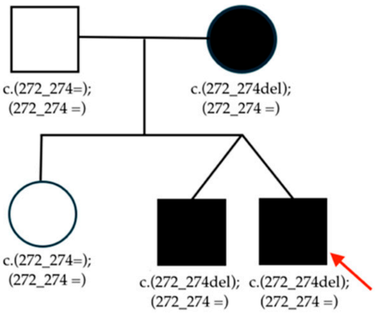

Family 2:

ID	Gene	Cataract surgery timing	Cataract phenotype	Ocular and/or systemic abnormalities	2° glaucoma	VA (Snellen)
1-2	MAF	Not reported	Nuclear	High myopia	no	Not reported
2-1	MAF	RE: 3m<br>LE: 1m	Nuclear	High myopia, MO	yes (BE)	RE: 0.05<br>LE: 0.4
2-2	MAF	BE: 1.5m	Nuclear, ant. polar	High myopia, MO	no	RE: 0.63<br>LE: 1.25

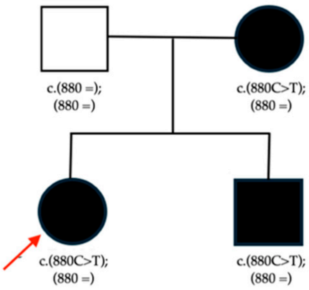

Family 3:

ID	Gene	Cataract surgery timing	Cataract phenotype	Ocular and/or systemic abnormalities	2° glaucoma	VA (Snellen)
1-2	EPHA2	6y	Not reported	N/A	no	Not reported
2-1	EPHA2	1.5m	Dense posterior	N/A	no	BE: FF

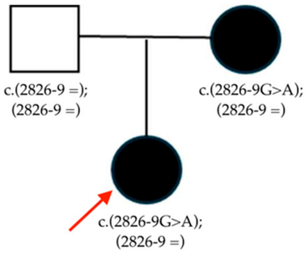

Family 4:

ID	Gene	Cataract surgery timing	Cataract phenotype	Ocular and/or systemic abnormalities	2° glaucoma	VA (Snellen)
1-2	GJA8	Not reported	Total	N/A	Not reported	Not reported
2-1	GJA8	BE: 1m	Total	N/A	yes (BE)	RE: 1.0<br>LE: CF 0.5m

2-2	GJA8	RE: 2m<br>LE: 1m	Total	N/A	yes (BE)	BE: 0.8

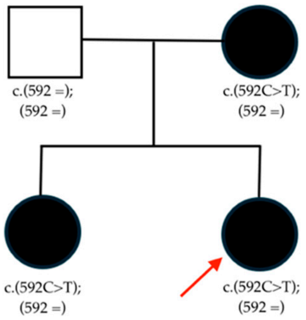

Family 5:

ID	Gene	Cataract surgery timing	Cataract phenotype	Ocular and/or systemic abnormalities	2° glaucoma	VA (Snellen)
2-3	CRYGC	BE: 1m	Nuclear	N/A	yes (RE)	RE: 0.12*<br>LE: 0.1*

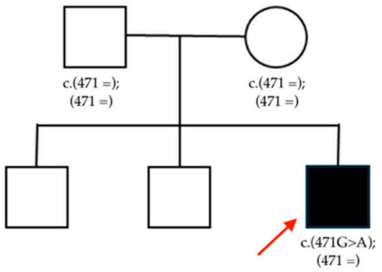

Family 6:

ID	Gene	Cataract surgery timing	Cataract phenotype	Ocular and/or systemic abnormalities	2° glaucoma	VA (Snellen)
2-2	MIP	BE: 1.5m	Nuclear	N/A	no	BE: 0.8

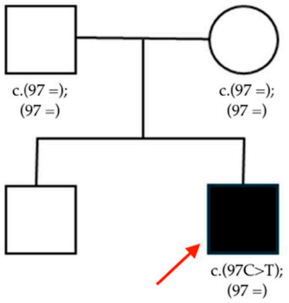

Family 7:

ID	Gene	Cataract surgery timing	Cataract phenotype	Ocular and/or systemic abnormalities	2° glaucoma	VA (Snellen)
1-1	GJA8	3m	Not reported	High astigmatism	no	RE: 0.8<br>LE: 0.7
2-2	GJA8	1.5m	Cortico-nuclear	N/A	yes (BE)	BE: FF

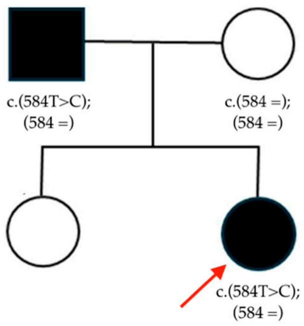

Family 8:

ID	Gene	Cataract surgery timing	Cataract phenotype	Ocular and/or systemic abnormalities	2° glaucoma	VA (Snellen)
2-2	CRYAA	RE: 2m<br>LE: 1.5m	Nuclear	Post. Syn., IA	yes (BE)	RE: 0.3<br>LE: 0.1

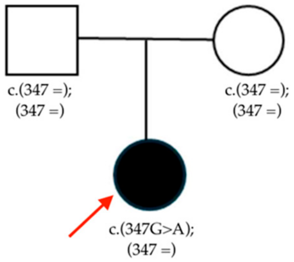

Family 9:

ID	Gene	Cataract surgery timing	Cataract phenotype	Ocular and/or systemic abnormalities	2° glaucoma	VA (Snellen)
1-2	NHS	BE: 30y	Not reported	Not reported	no	Not reported
2-2	NHS	BE:61y	Nuclear	N/A	no	RE: 0.4<br>LE: 1.0
2-3	NHS	No surgery	No cataract	N/A	N/A	Not reported
3-2	NHS	BE: 3m	Not reported	teeth malformation	no	BE: 0.8
4-2	NHS	BE: 1.5m	Dense posterior	MIC	no	BE: FF

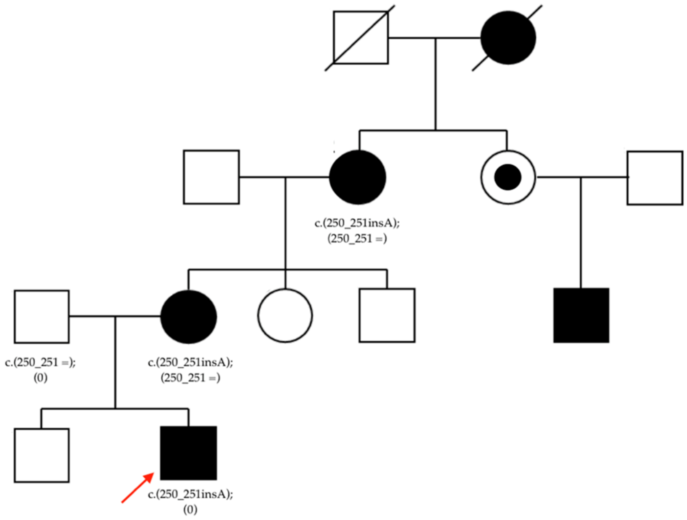

Family 10:

ID	Gene	Cataract surgery timing	Cataract phenotype	Ocular and/or systemic abnormalities	2° glaucoma	VA (Snellen)
1-2	NHS	BE: 30y	Not reported	N/A	no	Not reported

2-2	NHS	RE: 20y<br>LE: 27y	Nuclear	N/A	no	RE: 0.6<br>LE: 0.8
2-3	NHS	No surgery	No cataract	N/A	N/A	Not reported
3-1	NHS	RE: 1y9m<br>LE: 2y3m	Nuclear	N/A	no	BE: 1.25
3-2	NHS	No surgery	Nuclear	N/A	N/A	BE: 0.36 (LEA)

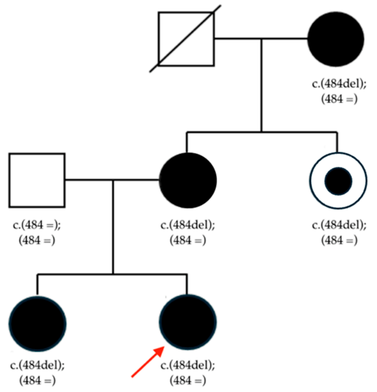

Abbreviations used in tables and pedigrees sorted alphabetically:

BE: both eyes; CF: Counting fingers; FF: Fixating and following; IA: iris atrophy; LE: left eye; LEA: single optotypes; MIC: microcornea; MO: microphthalmia; NHS: Nance-Horan Syndrome; n/a: not applicable; Post.Syn.: posterior synechiae; RE: right eye

Table S1. Clinical case summary of and variant correlation in solved CC patients.

Subjects		Gene Variant	Cataract Phenotype	Other Eye Anomaly	Co-existing Conditions	Synd. Traits	Cataract Surgery (timing)	Sequelae (IDx timing)	Sequelae treatment (timing)	VA (Snellen)	Age
f1	2-2♂ mtw1	*CRYBA1*	**BE:** Nucl.	N/A	PTB, PA	N/A	**BE:** LTX, IOL impl., PC, core ppV (1y9m).	**RE**: PCOs (2y2m). **LE:** Ant. Syn. (2y2m); 2°convergent strabism (4y).	**RE:** PCO removal (2y2m); OT (2y-8y). **LE:** Syn. removal at (2y2m).	**RE:** 0.63 **LE:** 0.4	15y
	2-3♂ mtw2						**BE:** LTX, IOL-impl, PC, core ppV (10m).	**BE:** PCO (1y2m); 2°strabism alterans (5y). **LE:** SG (6y).	**BE:** core ppV, PCO removal, Re-PC (1y2m). **LE:** PLEDs (6y); strabism surgery (11y).	**RE:** 1.0. **LE:** 0.5	
f2	2-1♀	*MAF*	**BE:** Nucl.	**BE:** MO, myopia	Mild MH	N/A	**RE:** LTX, ppV, PC (3m) **LE:** LTX, ppV, PC (1m).	**RE:** uncontrolled SG, ant. Syn. (3m). **LE**: SG (4y9m), PCO (14y3m).	**RE**: PLEDs (3m); TE MMC, PI (1y3m); bleb needling (1y5m); 2nd TE MMC, PI (3y10m); ant. ppV, Ahmed-Valve Impl. (4y9m); 2nd bleb needling (5y1m); 3rd bleb needling (5y5m), 4th bleb needling (7y); 2nd Ahmed Valve Impl. (7y3m); CPC (8y); 2nd CPC (10y5m); 3rd CPC (13y); 4th CPC (14y4m); 5th CPC (14y7m); Ahmed revision (11y3m); XEN DI (13y10m); Micropulse-CPC (14y1m); 5th bleb needling (15y6m); Bleb revision (15y7m). **LE**: PLEDs (4y9m); CPL (14y1m); ppV, PI, Baerveldt-DI (14y3m); ppV, PCO removal, Baerveldt-DI replacemet (14y4m); Baerveldt stent removal (14y4m); Baerveldt revision (14y6m); Express DI (14y8m); 2nd Express DI (14y11m).	**RE:** 0.05 **LE:** 0.4	21y
	2-2♂		**BE:** Nucl., ant. polar				**BE:** LTX, ppV, PC (1.5m).	**RE:** Convergent micro-strabism (4y11).	**LE:** OT (4y-10y).	**RE:** 0.63 **LE:** 1.25	20y
f3	2-1♀	*EPHA2*	**BE:** Dense post.	N/A	Muscular VSD, ASDII, mild PPS	N/A	**BE:** LTX, ant. ppV, PC (1.5m).	N/A	N/A	**BE:** FF	2y
f4	3-1♀	*GJA8*	**BE:** Total	N/A	N/A	N/A	**BE:** LTX, ant. ppV, PC (1m).	**BE:** PCO (4m). **RE:** SG (2y). **LE:** SG (4m);. 2°exo-, hypoertropia (5y).	**RE:** PLEDs (2y);. TE MMC (4y2m); OT (5y-8y). **LE:** PLEDs (4m); ICD, GTT (10m); ECD (11m); TE MMC (1y).	**RE:** 1.0 **LE:** CF 0.5m.	28y
	3-2♀						**RE:** LTX, ant. ppV, PC (2m). **LE:** LTX, ant. ppV, PC (1m).	**RE:** PCO, post. Syn. (1y). **BE:** SG (3y3m).	**RE:** PCO-, Syn. removal (1y1m). **BE:** PLEDs (3y3m);. **LE:** TE MMC (10y1m).	**BE:** 0.8	26y

Subjects		Gene Variant	Cataract Phenotype	Other Eye Anomaly	Co-existing Conditions	Synd. Traits	Cataract Surgery (timing)	Sequelae (IDx timing)	Sequelae treatment (timing)	VA (Snellen)	Age
f5	2-3♂	*CRYGC*	**BE:** Nucl.	N/A	N/A	N/A	**BE:** LTX, ppV, PI (1m).	**BE:** Pendular nystagmus (3m). **RE:** SG (2m). **LE:** PCO (6m); 2°convergent strabism (4m).	**RE:** PLEDs (3m); TE MMC, ant. ppV (4m) **LE:** PCO removal (5m); Botox injection M. rect. medialis (1y1m).	**RE:** 0.12* **LE:** 0.1*	3y10m
f6	2-2♂	*MIP*	**BE:** Nucl.	N/A	N/A	N/A	**BE:** LTX, ant. ppV (1.5m).	**BE:** CLAK (7m).	**BE:** Antibiotic ED (7m).	**BE:** 0.8	13y
f7	2-2♀	*GJA8*	**BE:** Corti-conucl.	N/A	N/A	N/A	**BE:** LTX, ant. ppV, IOL Impl. (1.5m)	**BE:** SG, PCO (6m)	**BE:** PLEDs (6m); TE MMC (7m).	**BE:** FF	1y3m
f8	2-1♀	*CRYAA*	**BE**: Nucl.	**LE**: post. Syn., IA	N/A	N/A	**LE**: LTX, ant. ppV, PC (1.5m) **RE**: LTX, ant. ppV, PC (2m)	**BE**: post. Syn (2m); SG (4m); corectopia, rec. post. Syn, jerk nystagmus (4m). **RE:** PCO (4m); rec. post. Syn (1.5y) **LE:** ant. Syn (4m); 2°convergent strabism (1y).	**BE**: Syn. removal, Myd. ED (3m); 2nd Syn. removal (4m); 3rd Syn. removal, PI (4m); TE (8m); CPC (10m); 2nd TE, DS, PI (1y). **RE**: PCO removal (4m); Syn. removal (1.5y); OT (1y-10y) **LE**: PLEDs (2.5y)	**RE**: 0.3 **LE**: 0.1	12y
f9	4-2♂	*NHS*	**BE:** Dense post.	**BE:** MC	PTB; PFO.	NA	**BE:** LTX, ant. ppV, PI (1.5m).	**RE:** PCO (4m). **LE:** PCO (7m).	**RE:** PCO removal (4m). **LE**: PCO removal (11m).	**BE:** FF	1y11m
f10	3-1♀	*NHS*	**BE:** Nucl.	N/A	N/A	N/A	**RE:** LTX, ant. ppV, PC, IOL Impl. (1y9m). **LE:** LTX, ant. ppV, PC, IOL Impl. (2y3m).	N/A	N/A	**BE:** 1.25	8y10m
	3-2♀						N/A	N/A	N/A	**BE:** 0.63*	4y3m

*Abbreviations in alphabetical order: ASD: atrial septal defect; BE: both eyes; CLAK: contact lens-associated keratitis; CPC: cyclophotocoagulation; CPL: canaloplasty; DI: drainage implantation; DS: deep sclerectomy; ECD: external cyclodialysis; ED: eye drops; FF: fixating and following; GGT: goniotomy; IA: iris atrophy; ICD: internal cyclodialysis; IDx: initial diagnosis; IOL: intraocular lens; LE: left eye; LTX: lentectomy; MC: microcornea; MH: muscular hypotonia; MMC: mitomycin; MO: microphthalmia; mtw: monogenic twin; Myd.: mydriatic; Nucl.: Nuclear; OT: occlusion therapy; PA: perinatal asphyxia; PC: posterior capsulotomy; PCO: posterior capsular opacification; PI: peripheral iridotomy; PLEDs: pressure-lowering eye drops; PTB: preterm birth; ppV: pars plana vitrectomy; PPS: peripheral pulmonary stenosis; rec: recurrent; RE: right eye; Ren.: Renal; SG: secondary glaucoma; Syn: synechiae; TE: trabeculectomy; VA: Visual acuity; VSD: ventricular septal defect; *: Lea single optotypes*

Table S2. Cataract-relevant gene set applied in WES data analysis

AASS	*BUB1B*	*CRYGA*	*EZR*	*GPX1*	*LGSN*	*NECAP2*	*PEX3*	*RRAGA*	*TGFBI*
ABCA3	*CANX*	*CRYGB*	*FAM126A*	*GSTM1*	*LIM2*	*NECTIN3*	*PEX5*	*RRM2B*	*TMCO3*
ABCB6	*CAV1*	*CRYGC*	*FAR1*	*GSTT1*	*LMX1B*	*NEU1*	*PEX6*	*RYR1*	*TMEM70*
ABCD3	*CBS*	*CRYGD*	*FBN1*	*GTF2IRD1*	*LONP1*	*NF2*	*PEX7*	*SALL4*	*TNPO1*
ABHD12	*CC2D2A*	*CRYGS*	*FKRP*	*GUCY2D*	*LOXL3*	*NHS*	*PIGY*	*SC5D*	*TOR1AIP1*
ADAM9	*CCNP*	*CRYZ*	*FKTN*	*HCCS*	*LRP2*	*NOD2*	*PITX2*	*SCHIP1*	*TRAPPC11*
ADAMTS10	*CDC25B*	*CTDP1*	*FLNB*	*HIP1*	*LRP5*	*NR2E3*	*PITX3*	*SEC23A*	*TRNT1*
ADAMTS17	*CHD7*	*CTNND2*	*FOXE3*	*HMX1*	*LRP5L*	*NRCAM*	*PKN1*	*SIL1*	*TRPM3*
ADAMTS18	*CHMP4B*	*CYP27A1*	*FTL*	*HSF4*	*LSS*	*OAT*	*PNPT1*	*SIPA1L3*	*TUBA1A*
ADAMTSL4	*CLPB*	*CYP51A1*	*FYCO1*	*HSPG2*	*LTBP2*	*OCRL*	*POLG*	*SIX5*	*TUBB*
ADIPOQ	*CNBP*	*DHCR7*	*FZD4*	*IARS2*	*LTBP3*	*OGG1*	*POMGNT1*	*SLC16A12*	*TUBB2A*
AGK	*CNGB3*	*DMPK*	*GALE*	*IDO1*	*MAB21L2*	*OPA1*	*POMT1*	*SLC33A1*	*UCHL1*
ALDH18A1	*COL11A1*	*DNASE2B*	*GALK1*	*IFNGR1*	*MAF*	*OPA3*	*POMT2*	*SLC40A1*	*UNC45B*
ANK2	*COL18A1*	*DNM2*	*GALT*	*IKBKG*	*MAFA*	*OTX2*	*PORCN*	*SLC4A4*	*VCAN*
AP4B1	*COL2A1*	*DNMBP*	*GBA2*	*INPP5K*	*MAN2B1*	*P3H2*	*PQBP1*	*SLC7A8*	*VIM*
APP	*COL4A1*	*DOCK5*	*GCM2*	*INTS1*	*MED12*	*PANK4*	*PRDX5*	*SLURP1*	*VLDLR*
ARID1B	*COL4A2*	*DST*	*GCNT2*	*IPO13*	*MED13*	*PARK7*	*PROX1*	*SMO*	*VSX2*
ARSL	*COL4A5*	*DYNC1H1*	*GDF3*	*ITM2B*	*MIP*	*PAX6*	*PRX*	*SORD*	*WDR36*
ASPH	*COL7A1*	*EFNA5*	*GEMIN4*	*JAM3*	*MIPEP*	*PDE6B*	*PTCH1*	*SOX1*	*WDR87*
ATAD3A	*CPAMD8*	*EIF2B2*	*GFER*	*KCNA4*	*MIR184*	*PEX1*	*PTEN*	*SOX2*	*WFS1*
B3GLCT	*CRYAA*	*EPG5*	*GJA1*	*KCNAB1*	*MMP1*	*PEX10*	*PTH*	*SPARC*	*WNT3*
B4GALT7	*CRYAB*	*EPHA2*	*GJA3*	*KCNJ13*	*MVK*	*PEX11B*	*PXDN*	*SRD5A3*	*WRN*
BCOR	*CRYBA1*	*ERCC2*	*GJA8*	*KIAA1109*	*MYH9*	*PEX12*	*RAB3GAP1*	*STX3*	*XRCC1*
BEST1	*CRYBA2*	*ERCC6*	*GJB6*	*KLRG1*	*MYOC*	*PEX13*	*RECQL4*	*SUOX*	*XYLT2*
BFSP1	*CRYBA4*	*ERCC8*	*GLA*	*LARGE1*	*NACC1*	*PEX14*	*RGS6*	*TAF1A*	*YWHAE*
BFSP2	*CRYBB1*	*ESCO2*	*GNAS*	*LCA5*	*NAT8*	*PEX16*	*RIC1*	*TAPT1*	*ZNF350*
BIN3	*CRYBB2*	*ETFDH*	*GNPAT*	*LCT*	*NCOA6*	*PEX2*	*RNF149*	*TDRD7*	
BRD4	*CRYBB3*	*EYA1*	*GPR161*	*LEMD2*	*NDP*	*PEX26*	*RPE65*	*TFAP2A*	
